# Rugby and Shoulder Trauma: A Systematic Review

**Published:** 2014-09-01

**Authors:** R. Papalia, A. Tecame, G. Torre, P. Narbona, N. Maffulli, V. Denaro

**Affiliations:** (1) Department of Orthopaedic and Trauma Surgery, Campus Bio-Medico University of Rome, Via Alvaro del Portillo 200, Rome, Italy; (2) Department of Musculoskeletal Disorders, Faculty of Medicine and Surgery, University of Salerno, 84081 Baronissi, Salerno, Italy.; (3) Centre for Sports and Exercise Medicine, Barts and The London School of Medicine and Dentistry, Mile End Hospital, 275 Bancroft Road, London E1 4DG, England; (4) Departamento de Artroscopía, Sanatorio Allende, Independencia 757 1er Piso, Córdoba, Argentina

**Keywords:** rugby, shoulder injuries, Bankart lesion, SLAP lesion, arthroscopy

## Abstract

Rugby is a popular contact sport worldwide. Collisions and tackles during matches and practices often lead to traumatic injuries of the shoulder. This review reports on the epidemiology of injuries, type of lesions and treatment of shoulder injuries, risk factors, such as player position, and return to sport activities. Electronic searches through PubMed (Medline), EMBASE, and Cochrane Library retrieved studies concerning shoulder injuries in rugby players. Data regarding incidence, type and mechanisms of lesion, risk factors and return to sport were extracted and analyzed. The main reported data were incidence, mechanism of injury and type of lesion. Most of the studies report tackle as the main event responsible for shoulder trauma (between 50% and 85%), while the main lesions reported were Bankart lesions, Superior Labral tear from Anterior to Posterior (SLAP tears), anterior dislocation and rotator cuff tears. Open or arthroscopic repair improve clinical outcomes. Shoulder lesions are common injuries in rugby players. Surgical treatment seems to be effective in for rotator cuff tears and shoulder instability. More and better designed studies are needed for a higher Level of Evidence analysis of this topic.

## I. INTRODUCTION

Rugby is a worldwide sport. There is increasing interest in rugby traumatology areas given the high injury rate ^1^. Rugby is played by two teams of 15 or 7 players, divided in “forwards” and “backwards”, playing different roles. The main event of the game consists of the scrum, in which the forwards players of the two teams contest the ball. After the ball has been obtained by one of the teams, the main aim is to reach the end of the opponent field to score a try. The players of the other team try to stop this through tackling. Tackle represents the main risk-event for injuries in rugby matches ^2–6^. The clinical approach to collision sports medicine, should consider the physical features of a player, with high athleticism, BMI often above 25 (because of increase of the muscle lean mass, in relation to height), and a high pain threshold ^2^.

To define sport related clinical events, the incidence of injury is reported as injury events per number of playing hours and the severity of the damage is expressed as the number of matched missed by the hit player, since the injury. An injury is termed “severe” if it causes to miss five or more matches (16–30% of all injuries) ^1^.

Overall injury rate in a rugby league is of 40.3 injuries per 1000 player hours ^7^. Tackling accounts for 46–90% ^1^ of total injuries, and the injuries derived from tackling are caused by the violent collision among two or more athletes. Both the tackled and the tackling players can report severe injuries, but shoulder lesions are more likely in the ball-carrier, the tackled player, who puts the adducted arm on the front-side of contact as a shield-mechanism, exposing the joint to excessive collision forces, during tackle and when hitting the ground. Though tackle is the most important collision event, so the main risk factor for injury, not all the injuries are caused by contact events among players. Shoulder injuries occur often in the try-scoring phase, when the player dive forward to reach the try line with the ball-carrying hand. ^8^. Generally, shoulder injuries represent 28% of all injuries ^1^.

Studies ^2, 5, 9, 10^ concerning sport injuries in rugby players available in literature reported about epidemiology of collision events, analyzing type and percentage of incidence of different shoulder lesions, through retrospective case series and cohort studies. Studies ^11^ assessing the risk of development of direct-injury consequences have been published, next to therapeutic trials evaluating outcomes of surgical and clinical treatments for injuries. Furthermore, video-analysis studies have been carried out to define the exact mechanisms of injury, by reviewing videos of the collision events in matches ^8, 12–14^.

This investigation systematically reviews the available studies concerning shoulder injury in rugby players. The main focus is represented by incidence and percentage of different kind of lesions affecting the shoulder joint, causes and mechanisms of injury and therapeutic options. Furthermore, we try to ascertain whether the player role affected the injury rate and other risk factors are involved in trauma.

## II. METHODOLOGY

**Figure 1:**
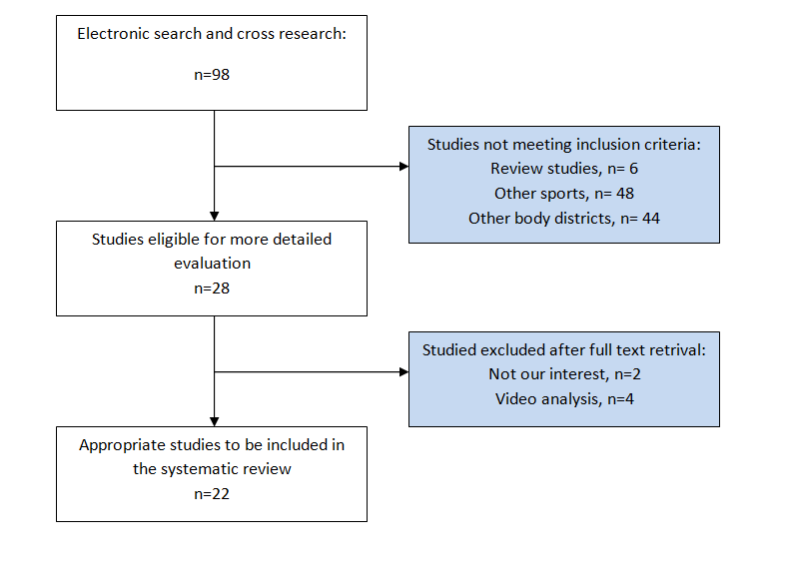


A first search was performed through online databases, to retrieve articles concerning the research topic. Medline, PubMed (http://www.ncbi.nlm.nih.gov/pubmed), Cochrane (http://www.thecochranelibrary.com/view/0/index.html) and Google scholar (http://scholar.google.it/) were searched until 25 June 2014. Search-terms and combination of these were: [Rugby AND shoulder], [Rugby AND shoulder AND injury], [Rugby AND shoulder AND slap OR lesion], [Rugby AND shoulder AND arthrosis], [Rugby AND shoulder AND surgery], [Rugby AND shoulder AND hagl], [Rugby AND shoulder AND instability], [Rugby AND shoulder AND rotator cuff]. All type of studies published in peer reviewed journals were firstly retrieved. Studies about shoulder trauma and concerning: mechanisms of lesion, injury incidence, therapeutic options, risks and consequences of injury were screened. After consulting the title and the abstract of the articles, only clinical randomized trials, prospective and retrospective cohort studies, cross-sectional studies on live human subjects were selected for the review, studies on cadavers were excluded. Of the studies eligible for inclusion, the whole bibliography was accurately screened, to identify studies missed through online searching. Taking into account the language capabilities of the reviewers, studies in English, Spanish, French and German have been read for further investigation. Of these, the full-text articles were carefully read and evaluated by two different reviewers (G.T. and R.P.) who discussed together whether a study should be included or not. All the cohorts enrolled by included studies were composed of not-professional to elite league rugby players. Relevant data reported by the studies were extracted and summarized into tables in order to provide reviewers with a systematic overview of the included papers. The studies inclusion process is shown in fig.1.

## III. RESULTS

### A. Number and type of studies

Twenty-two studies were included, meeting the inclusion criteria. Of these, 2 were cross sectional studies, 11 were cohort studies, 4 were observational studies and 5 were case series. The characteristics of the studies included are summarized in tab.1.

### B. Included studies data

A total of 4530 players were examined, with 3008 shoulder injuries reported. Studies included different level male rugby players (tab.1). Mechanism of injury was reported in 10 studies, main were: tackle, ruck and onto-arm fall. Seven epidemiological studies observed the incidence of injuries ^3, 5, 6, 15–18^. Therapeutical studies ^19–23^ assessed outcomes after arthroscopic surgery or open Latarjet procedure or capsulae reparation. Isokinetic testing was carried out in two studies ^24, 25^ and joint position sense in one ^26^. Five studies ^3, 14, 18, 19, 21^ reported recurrent injuries rate.

### C. Outcome measures

ROM was reported in three studies ^2, 19, 25^ (tab.2). Constant score and Oxford shoulder score were reported in one study ^23^. Rowe score was reported in two studies ^19, 22^ and Walch-Duplay score was reported by one study ^22^.

### D. Epidemiological studies

Lynch et al. ^18^ collect epidemiological data in South African Premier team rugby Union, about incidence of several lesion types as first, second or third injury, associating rates with play role. Dislocation, impingement and rotator cuff strain occurred more frequently as first injury (respectively n=8, n=3 and n=3), while rotator cuff tears had a grater incidence as second injury (n=7). Tackle represent the main injury event (61.5%). Primary injuries occurred more often in Centers (38.5%) and Tight Five (23.2%), as well as for second injuries. In the second study by Kawasaki et al. ^17^, 378 players were evaluated: in this cohort, 74 injuries were observed and 51.9% had a previous history of dislocation. The incidence reported was of 8.5 per 1000 played hours (PH) in matches and 0.2 per 1000 PH in practices. Results from observation of different level English rugby teams, revealed a total incidence of 243 injuries (all body districts) per 6186 player-match hours, with a rate of recurrent injuries of 11.5% in the study of Palmer-Green ^3^. Regarding shoulder injuries, a total of 6 Rotator Cuff tears and 8 dislocations were observed ^3^. Headey et al. ^27^ reported an incidence of 4.3/1000 PH of injuries which cause a missed match, assessing the higher percentage of incidence for acromioclavicular joint injury (32%). The main event causing injury was the tackle (65%) and backs showed an increased trend for tackle-derived injuries. Furthermore, Roberts et al. ^4^ reported an incidence of shoulder joint and ligament injuries of 1.7 per 1000 PH and an overall incidence of shoulder trauma of 2.3 1000 PH. An incidence of shoulder injuries of 3.1 injuries per 1000 athletic exposures, was reported by Usman and McIntosh ^6^, with a major involvement of acromioclavicular joint and then of the glenohumeral. Gabbett and Domrow ^16^ reported a shoulder injury rate of 4.2% in all body districts.

Concerning the correlation between injuries and play role, significantly increased shoulder injury rate occurs in Flankers (37 events per hour of play, 20.1% of overall incidence), five-eights (28 events per hour of play, 15.2% of overall incidence), while the lowest injury rate occurs in Half-backs (9 events per hour of play, 4.9% of overall incidence) ^5^. According to this study, Usman and McIntosh ^6^ observed an increased rate of injury in forwards, than in backs, as well as Kawasaki et al. ^17^, who reported an odds ratio of 1.8, and Bohu et al. ^15^, whose percentage of forward vs. backs was 55.6%. In this study ^15^ other factors were also related to injury: of these, the match phase relates with the position of the injured player (maul and ruck were injury events for forwards, OR 2.69) and injury rate relates with level of play (higher incidence in senior and junior players, p<0.001).

### E. Clinical evaluation of injuries

Cheng et al. ^11^ performed ultrasound analysis for assessment of shoulder laxity in 169 healthy players and 46 players with shoulder instability. A higher mean shoulder translation (in all directions) occurs in the normal shoulders of joint laxity affected subjects (p<0.05), compared with healthy subjects, independently from dominance.

Horseley et al. ^28^ assessed through EMG analysis whether a SLAP lesion could affect muscle groups activation before and during tackle. Results showed that Serratus Anterior was activated prior to all the other muscles, except for the infraspinatus, in non-affected shoulders (p<0.024). Serratus Anterior also activates earlier than the other muscles (except for latissimus dorsi), in shoulders with a SLAP lesion (p<0.03). Between subjects comparison showed that biceps activation was delayed in SLAP shoulders (p<0.01), while no difference occurred for other muscles (p>0.05).

Kawasaki et al. ^10^ evaluated the “rugby shoulder” through computed tomography osteoabsorptiometry in 25 athletes, compared with 17 healthy controls. Signal intensity (assessed in Hounsfield Units, HU) was higher in subjects than controls, thus revealing a higher mechanical loading of the whole shoulder.

### F. Bio-mechanical testing

Internal and external rotation strength of the shoulder was assessed through isokinetic testing in two studies ^24, 25^. In the study of Edouard et al. ^24^, the cohort was compared with non active subjects, ANOVA demonstrated significant correlation between rugby practice and increased peak torque in both intrarotation (IR) and extrarotation (ER) (p<0.05). McDonough and Funk ^25^ compared injuried and non-injuried players; IR was significantly greater in non-injuried players (p=0.02).

Horsley et al. ^9^ evaluated rugby players posture in comparison to soccer players. Significant difference occurs in terms of active range of movement (ROM), while no difference was observed concerning shoulder parameters such as Glenohumeral Internal Rotation Deficit (GIRD) and posterior capsular tightness. One study ^26^ assessed shoulder joint position sense (JPS) in players with SLAP, dislocation or anterior instability, compared with healthy controls. JPS testing consisted in assessing success in external rotation tasks (45° and 80°), from a position of 90° abduction in supine lay. Within subjects assessment showed no significant difference in terms of absolute error score depending on limb side (p=0.98), while testing angle affected significantly absolute error (p=0.002). Comparison between groups showed significantly higher JPS in controls than in injured players. If only the right side was considered, the testing angle affected significantly the absolute error score (p=0.002).

### G. Therapeutic studies

Bonnevialle et al. ^19^ presented outcomes by reviewing retrospectively 35 cases of open surgery for instability with a minimum 5 years follow up. Thirty-two of 33 subjects returned to play rugby at same level before surgery. A second injury occurred in 17% of players, at an average of 3.8 years since surgery. ROM was compared with the controlateral limb and was decreased (p<0.05), as was the subscapular muscle force, assessed by dynamometry (2.05Kg of difference between affected and not-affected limbs p<0.05). According to Rowe score, 86% of the shoulders had an excellent outcome. Imaging assessment on 22 patients revealed no sign of arthritis in 32% and grade I arthritis in 45%.

Arthroscopy outcomes were presented by three studies ^20, 21, 23^. Tambe et al. ^23^ presented a retrospective analysis of rotator cuff arthroscopic repair in 11 players. The mean Constant score improved post-operatively (44 to 99), as well as Oxford Shoulder score (34 to 12). Players returned to sport activities in a mean time of 4.8 months (only one retired for personal reasons). Funk and Snow ^20^, showed that SLAP lesion treated by arthroscopy have a high success rate (94% of patient returned to before surgery physical performance at 6 months from surgery). Time to return to physical activity was between 2.6 (isolated SLAP) and 6 months (anterior and posterior glenoid injury with SLAP). Larrain et al. ^21^ presented Bankart lesion arthroscopic or open Latarjet treatment outcomes, assessing that open surgery was required more in recurrent instability group. Results are comparable in term of success in acute or recurrent instability (84.6% vs. 86.7% had excellent outcome, 10.2% vs. 4.9% had good outcome). All subjects with acute instability returned to same level activity in a mean time of 5.3 months, while return to sport was of 84.3% in the recurrent instability group. The open Latarjet-Patte was also retrospectively evaluated by Neyton et al. ^22^, with an assessment through imaging and functional tests at 5 years follow-up. Radiographic results showed that bone healing occurred in 89% of the cases. At final follow-up, 70% of shoulders were free from arthritis and 30% presented grade 1 arthritis. No recurrence was noted. Sixty-five percent of patients returned to sport activity after the procedure between 3 to 24 months. The mean Walch-Duplay score was 86 points, and the mean Rowe score was 93 points.

## IV. DISCUSSION

The great interest of sports medicine in rugby is due to an increased risk of severe musculoskeletal injuries during matches and training of this contact sport, spread all over the world. Several studies have been carried out in order to evaluate injury rates, mechanisms of injury, therapeutic options and return to sport activities. This review collected evidence concerning shoulder injuries, about incidence of different shoulder lesions and therapeutic chances to let the player back to sport.

Bankart lesions ^11, 21^, SLAP ^2, 20, 28^, dislocation and rotator cuff tears ^18^ (resulting in acute or chronic instability) are the main lesions occurring during the games. Generally, tackle is the main injury-related event ^3–6, 9, 19, 20^, reported between 50% ^3^ and 85% ^20^. Therapeutic studies evaluated the outcomes of open and arthroscopic surgery, with return to sport in 96% of patients for open surgery ^19^ and between 84.6% ^21^ and 94% ^20^ in the mean time to return to sport between 2.6 and 6 months ^20^. All the clinical scores evaluated improved after surgery.

From an overall prospective shoulder, compared to other body districts ^29^, is not the main involved site, but the increased trend of shoulder lesion in rugby ^29^ turns the attention of orthopaedics and sports medicine surgeons on these invalidating lesions. Funk and Snow ^20^ reported that the main mechanism of injury during tackle is the direct shoulder collision with the opponent or the fall onto the abducted arm, resulting in a stretching and strain of the ligaments ^20^. To these mechanisms, Crichton et al. ^8^ added the “try scorer” mechanism, which consists in the fall of the player on the flexed arm in the phase of try, putting the ball beside the try line. However, independently from the mechanism, main injuries seem to be Bankart lesions, SLAP and rotator cuff tears ^8^.

The video analysis studies ^8, 12–14^, correlate the play event (tackle, ruck) to the mechanism of injury and type of lesion. King et al. ^12^ reported an increased rate of injury for tackler compared to the tackled player in the second half of the matches and it is probably a result of the overload that repetitive collisions bring to the shoulder. Rate of shoulder lesions reported by video analysis is 13.6% of all the injuries ^13^.

Significant evidence was reported about the role of players correlated to shoulder injuries. Sundaram et al. ^5^ stratified the cohort by role and observed that an increased injury rate occurred in flankers and five-eights. Accordingly to Usman and McIntosh, who reported an increased rate in forwards, in comparison to backwards. The primary reason for these findings is that forwards are mainly involved in high physically demanding activities, such as tackles, rucks and sprint and that they are involved in higher rate of tackle per match ^5^. In contrast with these opinions, Headey et al. ^27^ reported an increased rate for backwards in tackle-derived shoulder injuries ^27^. Furthermore a statistically significant correlation was found between play level and rate of shoulder injury ^6^.

Apart from game-play contact, some other risk factors have been identified. Cheng et al. ^11^ reported analysis of laxity of the shoulder, assessing that instability correlates with an increased translation of the shoulder in biomechanical testing ^11^. This may be a significant risk factor for recurrent dislocation of the joint.

Regrettably, the methodological quality of the studies included in this review, is poor. The studies included have a Level of Evidence (LOE) between II and IV, therefore no critical appraisal can be utilized in order to assess the quality. Though the studies reported significant results and cohort are actually large, no I level study can be carried out for this topic, because of the casual involvement of the players in injuries. Moreover, the different kind of data reported and way of presentation, do not allow an overlap of the findings and then a statistical metanalysis.

### Strength and limitations

Among the strength points of this review, we can say that many studies evaluate together outcomes from different sports, while we preferred to report only outcomes concerning rugby, so that no confounding factors can be addressed to the different kind of collisions (e.g. for American football) or different bio-mechanics of the joint movements (for Over-head sports). Furthermore, only shoulder lesions have been taken into account, excluding studies which did not report data about shoulder. On the other hand, several limitations should be also considered. First of all, the lack of a high LOE of the included studies didn’t allow us to provide significant evidence about the topic. Moreover, the different design of the studies, prevent us to compare data and to perform an appropriate statistical analysis. In addition, we excluded studies in unknown languages which could even bring a significant contribution to the results.

## V. CONCLUSION

Shoulder lesions are reported in several studies as common injuries in rugby players, including severe damage to the soft tissues of this joint. Surgical treatment allows the player to be back to play sport in many cases, often at the same level than before the operation. Actually, no study reports data about rehabilitation, so that is not possible to evaluate the role of post-operative recovery protocol in the return to sport activities. However, the lack of high LOE publications shows the need of better designed and methodologically qualitative studies about this topic.

## Figures and Tables

**Table 1:** Injuries details

Study	N.er of players	N.er of injuried players	Type of injury	Mechanism or traumatic event type	Intervention	Complications	% of complicated injuries
Cheng et al. 2012	202	43	Bankart lesion, superior labrum anterior to posterior, Hill-Sachs lesion, or frank dislocation		US shoulder laxity assessment		
Edouard et al. 2009	33	0			Isokinetic testing for internal and external rotation of the shoulder		
Horsley et al. 2010	22	7	SLAP		EMG assessment of muscle group activation during tackle		
Horsley et al. 2012	50	0			Posture analisys compared with non over-head players (soccer)		
Horsley et al. 2013	87	87	SLAP (83%), Rotator cuff tears (43%), Bankart tear,	Direct takling (56%), Onto-the-arm fall (10%), Not-specified (30%)	Shoulder arthroscopy		
Lynch et al. 2013	95	95	Dislocation, Impingement, Rotator cuff tear, Subluxation, inflammation of shoulder girdle		Observation and classification through modified Orchard Sports Injury Classification System	Recurrent injuries (8 players)	8.4%
McDonough et al. 2014	22	8			Isokinetic shoulder strength tests and ROM assessment		
Palmer-Green et al. 2013	0	243	All sites injuries	Being tackled (30%), tackling (20%),	Observation	Recurrent injuries (28)	11.5%
Roberts et al. 2013	0	0	All sites injuries	Contact events (80%)	Observation	Recurrent injuries	18%
Sundaram et al. 2011	269	166	Anterior instability	Tackling (66.3%), Onto-the-arm-fall (13%), Ruck (7.6%)	Observation		
Usman and McIntosh 2012	1475	606	Upper limb injuries	Being tackled, tackling, Overuse, Ruck	Obsevation		
Herrington et al. 2010	30	15	Anterior instability (45%), SLAP (30%), Dislocation (30%)		ROM assessment and joint position sense analysis		
Bonnevialle et al. 2008	33	33	Anterior instabilty	Tackle (50%)	Open capsule reparation, 5 years clinical and radiological assessment	Recurrent instability	17%
Funk and Snow 2007	51	18	single SLAP (61%), SLAP and Bankart lesion (17%), double SLAP and posterior labral lesion (11%)	Tackle	Arthroscopy		
Larrain et al 2006	198	198	Bankart lesion (90%), Bony defects, Capsular laxity, HAGL	Fall (55%), tackle (30%),	Arthroscopy or open modified Latarget procedure	Recurrent instability	79%
Neyton et al. 2012	34	34	Recurrent anterior instability	Tackle (35%), scrum (8.8%), other (38.2%)	open Latarget-Patte procedure		
Tambe et al. 2009	11	11	Rotator cuff tears and Bankart lesion or posterior labral tear	Abduction-external rotation impact (25%), Adduction-internal rotation impact (55%)	arthroscopic rotator cuff repair		
Headey et al. 2007	0	0	Hematoma (12%), Dislocation (14%), Acromioclavicular joint injury (32%), Rotator cuff injury (23%)	Tackle (65%)	Observation		
Kawasaki et al. 2014	378	74	Dislocation (45.7),	Tackle (67.6%),	Observation		
Kawasaki et al. 2013	42	25	Anterior instability (100%)		TC osteoabsorptiometry of the shoulder		
Gabbett and Domrow 2005	153		Rotator cuff injury (2.1%), Dislocation (1.5%), shoulder strain (0.6%)		Observation		
Bohu et al. 2014	1345	1345	Dislocation/subluxation	Tackle (69%), ruck	Observation	Recurrent instability, fractures, acromioclavicular injury, nerve ingury	

**Table 2:** ROM evaluation

Study	External rotation(°)		Gleno-humeral abduction(°)		Internal rotation (°)		Flexion (°)	
Horsley et al. 2012			160				165	
McDonough et al. 2014	Inj.	Not inj.			Inj.	Not inj.		
	L= 83.6, R= 83.9	L= 80.8, R= 80.5			L= 36.6, R= 38	L= 47.8, R= 45.3		
Bonnevialle et al. 2008	91.6						176.5	
